# Early reduction of *Leishmania infantum*-specific antibodies and blood parasitemia during treatment in dogs with moderate or severe disease

**DOI:** 10.1186/s13071-016-1519-0

**Published:** 2016-05-10

**Authors:** Laia Solano-Gallego, Laura Di Filippo, Laura Ordeix, Marta Planellas, Xavier Roura, Laura Altet, Pamela Martínez-Orellana, Sara Montserrat

**Affiliations:** Departament de Medicina i Cirurgia Animals, Facultat de Veterinària, Universitat Autònoma de Barcelona, Bellaterra, Spain; Hospital Clínic Veterinari, Universitat Autònoma de Barcelona, Bellaterra, Spain; Vetgenomics, Edifici Eureka, PRUAB, 08193 Bellaterra, (Barcelona) Spain

**Keywords:** Dog, *Leishmania infantum*, Antibody levels, Parasitemia, Treatment, Follow-up

## Abstract

**Background:**

*Leishmania infantum*-specific antibodies are used extensively for the diagnosis and monitoring of treatment in canine leishmaniosis. Different views have been described for the measurement of *L. infantum* antibody levels for the monitoring of anti-leishmanial treatment. In addition, molecular techniques using blood are frequently employed in the clinical setting. However, there are not enough studies to prove the usefulness of PCR in diagnosis, treatment monitoring and in assessing the prognosis of the disease. The objectives of this study were to evaluate *L. infantum*-specific antibodies and blood parasitemia at the time of diagnosis and during treatment and to correlate these with the dog’s clinical status.

**Methods:**

Thirty-seven dogs were diagnosed and followed-up during treatment (days 30, 180 and 365). The treatment protocol consisted of a combination of meglumine antimoniate for one month and allopurinol for at least one year. *Leishmania infantum*-specific antibodies and blood parasitemia were assessed by an end point sera dilution ELISA and by real-time PCR, respectively.

**Results:**

The majority of dogs were classified as LeishVet stage II (moderate disease) at the time of diagnosis (86 %) and the rest as stage III. Results showed variable levels of specific antibodies at the time of diagnosis [median ± interquartile range (IQR): 1372 ± 8803 ELISA units (EU)]. Twenty-three seropositive dogs (64 %) were detected as PCR-positive at the time of diagnosis. Interestingly, a rapid significant antibody level reduction was observed by day 30 of treatment (median ± IQR: 604 ± 2168 EU). A continuing significant decrease of specific antibodies was also found at days 180 (median ± IQR: 201 ± 676 EU) and 365 (median ± IQR: 133 ± 329 EU) in association with clinical improvement. A significant blood parasitemia reduction was also observed at all time points studied. Mean parasites/ml ± SD were 19.4 ± 79.1 on day 0, 2.2 ± 11.7 on day 30, 0.9 ± 2.9 on day 180, and 0.3 ± 0.7 on day 365.

**Conclusions:**

This study reports a significant reduction of *L. infantum* antibodies measured by an end point sera dilution ELISA method after 30 days of treatment associated with clinical improvement. A low proportion of sick dogs with moderate disease were negative by blood real-time PCR at the time of diagnosis.

## Background

*Leishmania infantum* is a protozoan parasite that causes canine leishmaniosis. The dog is the main reservoir of this infection, which represents a public health problem as canine leishmaniosis is one of the most prevalent zoonotic diseases worldwide. In southern Europe, the seroprevalence of disease is estimated to be between 5–30 %, but it has also been shown that the real prevalence of infection is greater than that detected by serology [1].

The range of clinical presentations and immune responses developed by *L. infantum-*infected dogs is very wide and variable. *Leishmania infantum* infection in dogs can manifest as subclinical infection, as a self-limiting illness or a serious life-threatening disease [2]. The most common clinical signs of canine leishmaniosis are skin lesions [2, 3] and lymphadenomegaly [4]. Other common signs include weight loss and anorexia, muscle weakness and ocular lesions. Furthermore, in sick dogs, it is very important to assess renal function as chronic renal failure is the leading cause of death in animals with clinical leishmaniosis [3]. Some laboratory findings which can be suggestive of leishmaniosis are mild non-regenerative anemia, hyperproteinemia, hyperglobulinemia, hypoalbuminemia and persistent proteinuria [4].

The progression of the disease depends on the type of immune response that infected dogs develop. Dogs that present clinical disease have high levels of *Leishmania*-specific immunoglobulins (IgG mainly, IgA and IgM) and a decreased or absent cellular immune response [5, 6]. This strong humoral response is one of the main causes of the pathogenesis of disease, because of type II and III hypersensitivity reactions [7, 8]. Dogs that can develop a strong specific T cell-mediated immunity appear to be able to control the parasite and manifest limited disease severity [6].

Due to the high variability in clinical presentations and immune responses, the diagnosis of canine leishmaniosis is complex. It is therefore necessary to perform a complete physical examination, complete blood count, complete biochemistry profile, urinalysis and serum protein electrophoresis [2, 3]. High levels of antibodies are associated with a high parasitic burden and disease [9]. There are many techniques for the serological diagnosis of canine leishmaniosis. Qualitative techniques such as rapid serological tests only give a positive and negative result. In contrast, quantitative serological tests such as the immunofluorescence antibody test (IFAT) and the enzyme-linked immunosorbent assay (ELISA) which give an antibody level, are considered the most suitable tests for the diagnosis [10, 11] of clinical leishmaniosis due to their reliable diagnostic performance. Real-time PCR is commonly also used in the diagnosis of clinical leishmaniosis. But, the sensitivity of this technique depends on the type of sample used and the tissues that appear to be more sensitive are bone marrow, lymph node and skin [4]. Other less invasive sampling techniques such as blood testing are also frequently used in the clinical setting. However, there are not enough studies to prove its usefulness as a diagnostic technique, in treatment monitoring and in assessing disease prognosis [12, 13].

The treatment protocol and prognosis are set according to the clinical stage of the patient. Four clinical stages have been designated according to clinical signs, clinicopathological abnormalities and the level of specific antibodies [2]. The most common treatment is the combination of meglumine antimoniate and allopurinol or miltefosine and allopurinol for four weeks, followed by administration of allopurinol for at least six months - one year [2]. During treatment, it is necessary to regularly monitor the same parameters mentioned for the diagnosis and to evaluate the patient’s clinical evolution. Treatment may be stopped if a complete clinical recovery of the animal and a marked decrease in antibodies are observed (low positive or seronegative) [3].

The usefulness of studying the kinetics of specific antibodies to assess the clinical outcome and response to treatment of the patient is under discussion. Controversial results have been described regarding the usefulness of measuring *L. infantum* specific antibodies for treatment monitoring. Some studies have shown a significant slow decrease in the level of *L. infantum*-specific immunoglobulins that correlated with clinical improvement but antibodies remained detectable over a long period of time [14–16] while other studies argue that there is no correlation between antibody levels and clinical status and that antibody levels are not useful for treatment monitoring [17, 18]. In addition, it has been stated that measurement of antibody levels is meaningless before six months of treatment [2, 3].

The objectives of this study were to evaluate the kinetics of specific anti-*Leishmania* antibodies using a two-fold serial dilution ELISA and to compare it to blood parasitemia at the time of diagnosis and during treatment in dogs with clinical leishmaniosis (with clinical stage II or higher), to correlate antibodies and blood parasitemia with clinicopathological abnormalities and to evaluate their relationship with clinical improvement of the disease.

## Methods

### Dogs

Thirty-seven dogs with clinical leishmaniosis were enrolled at the time of their diagnosis from January 2014 to May 2015. The dogs were treated at different Catalonian veterinary centers: *Fundació Hospital Clínic Veterinari* (Bellaterra, Barcelona), *Hospital Ars Veterinaria* (Barcelona), *Hospital Mediterrani Veterinaris* (Reus, Tarragona) and *Consultori Montsant* (Falset, Tarragona). The diagnosis of canine leishmaniosis (day 0) was made based on the results of a physical examination, a complete blood count (System Siemens Advia 120), a biochemical profile including creatinine, urea, total proteins, ALT and total cholesterol (Analyzer Olympus AU 400), serum electrophoresis (Hydrasys), urianalysis with urinary protein creatinine ratio (UPC) and quantitative serology for the detection of *L. infantum-*specific antibodies by means of an in-house diagnostic ELISA [11]. All dogs presented medium to high antibody levels [11]. Cytological or histological evaluations with *Leishmania* immunohistochemistry of cutaneous or other lesions were also performed when needed [19]. Dogs were classified into clinical stages at the time of diagnosis as previously described [2]. Dogs were treated with a daily subcutaneous injection of meglumine antimoniate (80–100 mg/kg) for a month and 10 mg/kg BID of oral allopurinol for 12 months. The dogs were followed up at days 30 (*n* = 36), 180 (*n* = 37) and 365 (*n* = 29) during treatment. A full physical examination and laboratory tests described above were also performed during treatment monitoring visits. A signed informed consent was obtained from all owners. Residual samples from blood EDTA tube and serum were used in this study. Therefore, ethical approval was not needed.

### ELISA

#### Diagnostic ELISA

The in-house ELISA was performed on sera of all dogs studied as previously described [11] with some modifications. The samples were diluted to 1:800 in PBS-Tween containing 1 % dry milk and incubated in *L. infantum* antigen-coated plates (20 μg/ml) for 1 h at 37 °C. Then, the plates were washed three times with PBS-Tween and once with PBS and incubated with Protein A conjugated to horseradish peroxidase (Thermo Scientific, dilution 1:30000) for 1 h at 37 °C. After that, the plates were washed again as described above. The plates were developed by adding the substrate solution *o*-phenylenediamine and substrate buffer (SIGMA*FAST* OPD, Sigma Aldrich). The reaction was stopped with 50 μl of 2.5M H_2_SO_4_. Absorbance values were read at 492 nm by an automatic reader (ELISA Reader Anthos 2020). All plates included the serum from a sick dog with confirmed infection as positive control and serum from a healthy dog as a negative control and all samples were analyzed in duplicate. The result was quantified as ELISA units (EU) related to a positive canine serum used as a calibrator and arbitrarily set at 100 EU.

#### Two-fold serial dilution ELISA

All samples with an optical density (OD) equal or higher than three were studied using a two-fold serial dilution ELISA. Sera two-fold dilutions were started at 1:800 and continued for 9 to 11 further dilutions for all time points studied for each dog (days 0, 30, 180 and 365). All samples were analyzed on the same day and in the same ELISA plate to avoid variability [20]. The result was quantified as ELISA units (EU) related to a calibrator arbitrarily set at 100 EU, with an OD value of one at the 1:800 dilution. The mean values of the dilutions at which the optical density (OD) were close to one was chosen for the calculation of the positivity % using the following formula: (Sample OD/Calibrator OD) × 100 × dilution factor. Sera were classified as: very high positive, when having a positivity percentage equal or higher than 40000 EU; high positive, when having a positivity percentage equal or higher than 9000 EU and less than 40000 EU; medium positive, when having a positivity percentage equal or higher than 500 EU and less than 9000 EU; low positive, when having a positivity percentage lower than 500 EU and equal or higher than 100 EU; very low positive, when having a positivity percentage lower than 100 EU and equal or higher than 35 EU. Sera with percentage lower than 35 EU, were classified as negative. The cut-off was established at 35 U (mean + 4 SD of values from 80 dogs from non-endemic area) as previously described [11].

### Blood DNA extraction and *Leishmania* real-time PCR

Total DNA was extracted from EDTA whole blood using the DNA Gene extraction kit (Sigma Aldrich) following the manufacturer’s instructions with slight modifications. Forty μl of proteinase K solution were added to all samples. Four hundred μl of whole blood were used for all the samples. The other steps were performed as described in the protocol. Blood from a clinically healthy non-infected dog was used as a control for DNA contamination in every DNA extraction performed.

Real-time PCR (RT-PCR) was performed with an absolute quantification as previously described with minor modifications [12]. Briefly, PCR mix reaction was prepared with 4 μl of DNA, 10 μl of master mix (TaqMan® Fast Advanced Master Mix, Life Technologies), 1 μl of *Leishmania* primers and probes (Custom TaqMan® Gene Expression Assay, Life Technologies) or 1 μl of another type of assay primers and probes [Eukaryotic 18S rRNA Endogenous Control (VIC™⁄MGB Probe, Primer Limited)] and 5 μl of H_2_O. PCR reaction was performed in duplicates for each sample and for each target gene.

In order to verify that the PCR was done successfully, a positive control for *Leishmania*, a negative control from non-infected clinically healthy dog and a blank (well without DNA sample) were included in all the plates. PCR was carried out in a QuantStudio Flex™ 7 Real-Time PCR system (Life Technologies). Thermal cycling profile consisted of 50 °C for 2 min in order to activate the enzyme called amperase and 20 s at 95 °C followed by 40 cycles of 1 s at 95 °C and 20 s at 60 °C [12].

Absolute quantification was carried out by the interpolation of the unknown samples to the standard curve generated from a negative sample spiked with different quantities of *Leishmania* promastigotes. Depending on the value of parasitic load, the samples were classified as negative (0 parasites/ml), low positive (< 10 parasites/ml), medium positive (10–100 parasites/ml), high positive (100–1000 parasites/ml) or very high positive (> 1000 parasites/ml) [13].

### Statistical analysis

Statistical analysis was performed with the IBM® SPSS® Statistics software version 22. A descriptive study of the level of antibodies and blood parasitemia on day 0, 30, 180 and 365 was performed, and the medians were compared using a Wilcoxon Signed Rank test. The difference between level of antibodies in each clinical stage were studied using the Mann-Whitney U test. The correlation between the level of antibodies and blood parasitemia and the clinical data of dogs (UPC ratio, total proteins, albumin, beta and gamma-globulins, hematocrit and hemoglobin concentration) was studied by a Spearman’s correlation. A *P*-value < 0.05 was considered statistically significant.

## Results

### Dogs

Thirty-seven dogs with at least moderate disease were included. Twenty-one out of the dogs were males (57 %) and 16 females (43 %). Ten out of the 16 females and 18 out of the 21 males (28/37; 76 %) were sexually intact.

There was a wide range of pure breeds represented (*n* = 26; 70 %): the most frequent breeds were the Boxer (*n* = 3; 8 %), French Bulldog (*n* = 2; 5 %), German Shepherd (*n* = 2; 5 %) and Golden Retriever (*n* = 2; 5 %). Other breeds were only represented once. Eleven mixed-breed dogs (*n* = 11; 30 %) were also diagnosed.

The median age at diagnosis was 54 months (4.5 years), with a range from 9 months to 153 months (12.5 years).

Thirty-two out of the 37 dogs were classified at the time of diagnosis as being in stage II of leishmaniosis and having moderate disease (86 %) and five as being in stage III with severe disease (14 %). The stage II dogs were sub-classified in stage IIa (24/32; 75 %) and stage IIb (8/32; 25 %) based on the presence or absence of proteinuria.

### Serology

At the day of diagnosis (day 0), the median level of antibodies was 1372 EU, and a marked inter-individual variability was also observed (interquartile range -IQR- 8803 EU). This variability was observed even within a single clinical stage or substage: the median and IQR of antibody level in clinical stage II and III was 1333 ± 5943 EU and 13,786 ± 40,523 EU, respectively; the median ± IQR in substages IIa and IIb was 1066 ± 2632 EU and 7928 ± 12164 EU, respectively. The distribution was significantly different (Mann-Whitney U test:  *Z* = -2.132, *P* = 0.033) between substages IIa and IIb, but not between stages II and III.

**Fig. 1:**
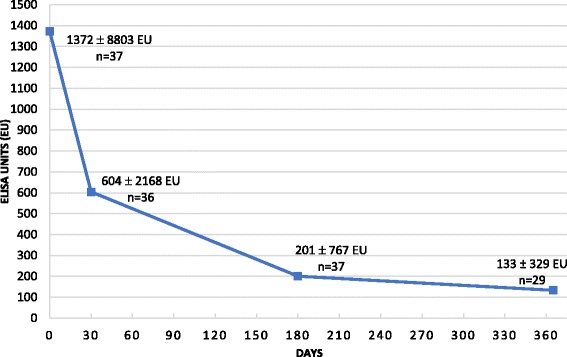
Results of the level of antibodies (median ± interquartile range) against *Leishmania infantum* at the time of diagnosis (day 0) and during the anti-*Leishmania* treatment* (days 30, 180 and 365) in 37 dogs with clinical leishmaniosis (at least stage II, moderate disease) **. *Anti-*Leishmania* treatment: meglumine antimoniate (100 mg/kg/SID/SC/30 days) combined with allopurinol (10 mg/kg/BID/PO/1 year). **Comparison between day 0 with the other days [day 30 (Wilcoxon signed-rank test: *Z* = -4.839, *P* < 0.0001), day 180 (*Z* = -4.895, *P* < 0.0001) and day 365 (*Z* = -4.703, *P* < 0.0001)]. Comparison between day 30 and day 180 (*Z* = -3.865, *P* < 0.0001), day 30 - day 365 (*Z* = -4.623, *P* < 0.0001) and day 180 - day 365 (*Z* = -4.335, *P* < 0.0001)

The kinetics of antibody levels is shown in Fig. 1. After thirty days of treatment a significantly marked decrease in the level of antibodies (Wilcoxon signed-rank test: *Z* = -4.839, *P* < 0.0001) was noted (median ± IQR: 604 ± 2168 EU). Antibody level continued to decline significantly after six months (Wilcoxon signed-rank test: *Z* = -4.895, *P* < 0.0001; median ± IQR: 201 ± 767 EU) and one year of treatment (Wilcoxon signed-rank test: *Z* = -4.703, *P* < 0.0001; median ± IQR: 133 ± 329 EU), although less markedly.

Despite the discernible reduction of the antibodies level in most of the dogs, only five became seronegative during the study period: three after six months of treatment (8 %) and two after one year (7 %). After one year of treatment, eight dogs (28 %) were classified as having a very low antibody level and twelve as having low levels (41 %) with only six dogs remaining with medium to high levels (21 %).

Parallel to the decrease in antibody levels, a clinical improvement including improvement in clinical signs and laboratory abnormalities was observed in all dogs after 30 days of treatment. At days 180 and 365 of treatment, the majority of dogs did not present any clinical signs. Forty-nine percent (18/37) and 65 % (19/29) of dogs no longer showed any change in the laboratory parameters at days 180 and 365, respectively. Some of the dogs remained only with mild proteinuria or mild hyperglobulinemia at that point.

Three dogs did not develop a reduced level of antibodies as expected. One had an increase in the level of antibodies at the day 180 visit (from 24,785 EU at day 30 to 28,564 EU at day 180), corresponding to a clinical relapse. The dog presented with moderate non-regenerative anemia, hyperproteinemia, hypoalbuminemia and increased beta and gamma-globulins. A cycle of treatment with meglumine antimoniate was repeated and molecular detection of *Hepatozoon* and *Babesia* as well as serological detection of *Ehrlichia*, *Anaplasma*, *Borrelia* and *Dirofilaria* were tested to rule out other concomitant parasitic and bacterial infections. Molecular and serological results were negative. At the next visit after repeating the treatment cycle, the level of antibodies had decreased (20,620 EU) although the laboratory parameters remained abnormal. Another case showed a slight increase in antibodies at day 180 control visit, but less marked [from 2484 EU (day 30) to 3453 EU (day 180)], in conjunction with a clinical relapse noted by the appearance of exfoliative dermatitis on the ears and elevated gamma-globulins. This dog probably needed a second course of treatment but the owner declined and was lost to further follow-up. In another dog, a progressive rise in antibodies at each control was observed (day 0: 2086 EU, day 30: 2263 EU; day 180: 8045 EU). This dog received half of the recommended dose of allopurinol. After repeating the one month cycle of treatment with meglumine antimoniate and correcting the dose of allopurinol, clinical findings improvement and antibody level decrease were clearly detectable (day 356: 789 EU).

### Clinicopathological data

#### Correlation of specific *L. infantum* antibody levels, blood parasitemia and clinical data at the time of diagnosis

The results of correlation of specific *L. infantum* antibody levels, blood parasitemia and clinical data are summarized in Table 1. Antibody levels  were positively correlated with the UPC ratio, the total protein level, the gamma-globulins and the blood parasitemia. The strongest correlation with the level of antibodies were observed for total protein (Spearman's correlation coefficient r_s_ = 0.698; *P* < 0.0001) and the level of gamma-globulins (r_s_ = 0.790; *P* < 0.0001). The antibodies were negatively correlated with the albumin, the hematocrit and the hemoglobin concentration. There was no correlation between the level of antibodies and the beta-globulins at diagnosis (r_s_ = 0.285; *P* = 0.102).

**Table 1:** Correlation between the level of antibodies, the clinicopathological alterations and the blood parasitemia at diagnosis

Parameter (units)	Spearman’s correlation coefficient (r_s_)	*P*-value
UPC ratio	0.412	0.021*
Total protein (g/dl)	0.698	< 0.0001*
Albumin (g/dl)	-0.381	0.020*
Beta-globulins (g/dl)	0.285	0.102
Gamma-globulins (g/dl)	0.790	< 0.0001*
Hematocrit (%)	-0.414	0.012*
Hemoglobin (g/dl)	-0.376	0.024*
Blood parasitemia (parasites/ml)	0.448	0.006*

UPC ratio, urinary protein creatinine ratio

*Statistically significant *P*-values

#### Kinetics of laboratory abnormalities during treatment

The dogs laboratory abnormalities evolved favorably during the treatment period (Table 2). The UPC ratio at day 0 was statistically higher than at the other time points of the study. The other parameters (total protein, albumin, gamma-globulins, hematocrit and hemoglobin) were statistically different between day 0 and the other time points of the study and between day 30 and the other time points of the study. The improvement observed between day 180 and day 365 was not statistically significant in all the parameters.

**Table 2:** Kinetics of laboratorial alterations at the time of diagnosis and during treatment. The median values ± interquartile range of laboratorial parameters at every point of the study

Parameters (units)	Reference intervals^g^	Day 0	Day 30	Day 180	Day 365
UPC ratio^a^	< 0.5	0.3 ± 0.7	0.2 ± 0.2	0.2 ± 0.5	0.1 ± 0.3
Total protein (g/dl)^b^	5.4–7.1	8.00 ± 3.05	7.08 ± 1.60	6.63 ± 0.67	6.70 ± 0.65
Albumin (g/dl)^c^	2.6–3.3	2.66 ± 0.89	2.72 ± 0.65	3.33 ± 0.54	3.36 ± 0.53
Gamma-globulins (g/dl)^d^	0.3–0.8	1.63 ± 3.03	0.91 ± 1.11	0.63 ± 0.28	0.61 ± 0.33
Hematocrit (%)^e^	40–61	36 ± 13	39 ± 10	44 ± 7	46 ± 9
Hemoglobin (g/dl)^f^	13.0–19.8	12.6 ± 4.2	13.7 ± 3.1	15.8 ± 2.6	16.3 + 4.0

^a^Comparison between day 0 and the other days [day 30 (Wilcoxon signed-rank test, *Z* = -2.987 *P* = 0.003), day 180 (*Z* = -2.521 *P* = 0.012), day 365 (*Z* = -2.500 *P* = 0.012)]. Comparison between day 30 - day 180 (*Z* = -0.456, *P* = 0.649), day 30 - day 365 (*Z* = -0.533, *P* = 0.594) and day 180 - day 365 (*Z* = -1.122, *P* = 0.262)

^b^Comparison between day 0 and the other days [day 30 (Wilcoxon signed-rank test: *Z* = -3.676, *P* = 0.000237), day 180 (*Z* = -3.784, *P* = 0.000155) and day 365 (*Z* = -3.579, *P* = 0.000345)]. Comparison between day 30 - day 180 (*Z* = -2.707, *P* = 0.007), day 30 - day 365 (*Z* = -2.033, *P* = 0.042) and day 180 - day 365 (*Z* = -0.205, *P* = 0.83)

^c^Comparison between day 0 and the other days [day 30 (Wilcoxon signed-rank test: *Z* = -2.040, *P* = 0.041), day 180 (*Z* = -5.004, *P* < 0.0001) and day 365 (*Z* = -4.703, *P* < 0.0001)]. Comparison between day 30 - day 180 (*Z* = -4.342, *P* < 0.0001), day 30 - day 365 (*Z* = -3.530, *P* < 0.0001) and day 180 - day 365 (*Z* = -0.573, *P* = 0.566)

^d^Comparison between day 0 and the other days [day 30 (Wilcoxon signed-rank test: *Z* = -4116, *P* < 0.0001), day 180 (*Z* = -4.357, *P* < 0.0001) and day 365 (*Z* = -2.084, *P* = 0.037)]. Comparison between day 30 - day 180 (*Z* = -3.752, *P* = 0.000175), day 30 - day 365 (*Z* = -4.065, *P* < 0.0001) and day 180 - day 365 (*Z* = -0.379, *P* = 0.705)

^e^Comparison between day 0 and the other days [day 30 (Wilcoxon signed-rank test: *Z* = -2.655, *P* = 0.008), day 180 (*Z* = -4.379, *P* < 0.0001) and day 365 (*Z* = -3.977, *P* < 0.0001)]. Comparison between day 30 - day 180 (*Z* = -2.657, *P* = 0.008), day 30 - day 365 (*Z* = -3.322, *P* = 0.020) and day 180 - day 365 (*Z* = -1.202, *P* = 0.229)

^f^Comparison between day 0 and the other days [day 30 (Wilcoxon signed-rank test: *Z* = -2.806, *P* = 0.005), day 180 (*Z* = -4.243, *P* < 0.0001) and day 365 (*Z* = -3.521, *P* = 0.000431)]. Comparison between day 30 - day 180 (*Z* = -3.181, *P* = 0.001), day 30 - day 365 (*Z* = -3.124, *P* = 0.002) and day 180 - day 365 (*Z* = -1.384, *P* = 0.166)

^g^UAB hematological and biochemical laboratory reference intervals

### PCR

PCR of blood was capable of detecting only 64 % (23/36) of the dogs as positive at diagnosis when compared with the quantitative ELISA. Overall, dogs had medium or low parasitemia. Only two were classified as high positive and none exceeded 1000 parasites/ml. Variability observed in parasitemia was also high: at day 0 the mean parasitemia was 19.43 parasites/ml with a standard deviation (SD) of 79.09 parasites/ml.

**Fig. 2:**
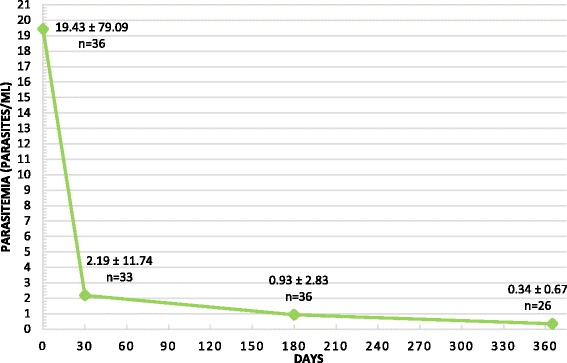
Results of the *Leishmania infantum* parasitemia (mean ± standard deviation) at time of diagnosis (day 0) and during the anti-*Leishmania* treatment* (days 30, 180 and 365) in 37 dogs with clinical leishmaniosis (at least stage II, moderate disease)**. *Anti-*Leishmania* treatment: meglumine antimoniate (100 mg/kg/SID/SC/30 days) combined with allopurinol (10 mg/kg/BID/PO/1 year). **Comparison between day 0 with the other days [day 30 (Wilcoxon signed-rank test: *Z* = -3.180, *P* = 0.001), day 180 (*Z* = -3.257, *P* = 0.001) and day 365 ( *Z* = -3.059, *P* = 0.002)]

The kinetics of *L. infantum* parasitemia is graphically represented in Fig. 2. The value of parasitemia at day 0 was significantly higher than those observed by day 30 (Wilcoxon signed-rank test: *Z* = -3.180, *P* = 0.001), day 180 (Wilcoxon signed-rank test: *Z* = -3.257, *P* = 0.001) and day 365 (Wilcoxon signed-rank test: *Z* = -3.059, *P* = 0.002).

The kinetics of PCRs values followed a similar trend as the levels of antibodies, with a rapid drop in average value at day 30 (mean ± SD: 2.19 ± 11.74 parasites/ml), which continued progressively during the rest of the treatment period reaching values close to zero (day 180: 0.93 ± 2.83 parasites/ml; day 365: 0.34 ± 0.67 parasites/ml). Despite the general reduction of the blood parasitemia, some dogs previously negative became low positive at year follow-up (*n* = 9; 42 %), without a clinical relapse. In addition, the three dogs that manifested clinical relapse during treatment (see above) showed an increase in the blood parasitemia parallel to the increase of antibodies: a two-fold increase in the first case and an increase from negative to low positive in the other two cases.

One dog showed a 15-fold increase in blood parasitemia at six months, not related to a clinical relapse. In another case, a slight and progressive increase of the parasitemia was observed starting from 30 days of treatment, but clinically this dog had improved as expected.

## Discussion

The present study showed that after 30 days of treatment there was a marked significant decline in the level of *L. infantum*-specific antibodies corresponding with clinical improvement as reported earlier in one study [21]. In previous studies, some authors found no correlation between clinical improvement of dogs studied and the level of antibodies and, therefore, they did not consider serology a useful parameter for treatment monitoring [17, 18]. Others argued that serology is not useful in the short term, because in the first weeks of treatment, serology does not correlate with the clinical course [22, 23]. The current recommendation about quantitative serology for treatment monitoring was to perform serology after six months of treatment [2, 3], due to the difficulty in detecting any clear reduction before. With the serial dilution method ELISA described here, it was possible to evaluate the treatment efficacy very early.

In line with observations by other studies [14, 16, 24], the antibody level continued to decrease progressively but less pronouncedly throughout the treatment period. As shown in previous studies [5, 25], dogs who became seronegative during the first year of treatment were a minority, but almost all of them reached much lower levels of specific antibodies when compared to the time of diagnosis.

Our study highlights the high dog inter-individual variability in the level of antibodies at the time of diagnosis, in agreement with another previous study [16]. This variability was evident even between dogs classified in the same clinical stage or substage.

We corroborated that monitoring the antibody kinetics is very useful for detection of clinical relapse of dogs under treatment since they are associated with an increased antibody level in blood [2, 3, 16, 25]. Relapsing clinical cases observed in this study were mainly due to a failure of treatment compliance leading to an inappropriate anti-*Leishmania* treatment protocol. However, one dog did not improve with adequate conventional treatment suggesting a very susceptible patient or a possible allopurinol drug resistance as recently reported [26]. This dog improved clinically with addition of meglumine antimoniate treatment in combination with allopurinol.

Our results show that the blood PCR technique has less diagnostic value than quantitative serology, 36 % of dogs with at least moderate disease [2] included would not have been detected based only on blood PCR. This finding is in agreement with other authors [10] as well as with the facts that it is well known that blood parasitemia might be intermittent [27] and the blood *Leishmania* parasite load is much lower than the load found in other tissues such as bone marrow in dogs with clinical leishmaniosis [10, 28, 29]. However, these results contrast with those observed by other studies [30, 31] who consider blood PCR more useful for diagnostic purposes than serology. In agreement with the results of this study, blood PCR is recommended to be used always accompanied by quantitative serology test and other diagnostic techniques in order to interpret the results but never as the sole diagnostic technique [3].

We observed a significant decline in the blood parasite load during the first 30 days of treatment and a progressive reduction throughout the remainder of the period, consistent with previous studies [12, 13, 30]. However, as previously described [32, 33], after long periods of treatment, low levels of parasitemia might be observed. The presence of *L. infantum* DNA was detected in some dogs in the present study after negative follow-ups. This could be due to the existence of intermittent parasitemia, also described by other authors [27]. It is also important to highlight that the presence of *L. infantum* DNA in the blood during the last follow up dates (days 180 or 365) was commonly not associated with a clinical relapse based on increased antibody levels, clinical signs and/or clinicopathological abnormalities. Despite that, two out of three dogs with clinical relapse became blood PCR positive. Therefore, blood real-time PCR during treatment monitoring should always be accompanied by a full physical examination, quantitative serology and routine laboratory tests. In addition, it is important to point out that in the majority of these treated dogs, clinical cure exists while parasitological cure does not as previously reported [32, 33]. It is also likely that long term treatment with allopurinol will induce parasite drug resistance as elegantly documented in natural clinical leishmaniosis in dogs [26].

A significant correlation was found between common clinicopathological abnormalities observed and antibody levels as previously reported [9, 34, 35]. In addition, a significant positive correlation was also noted between antibody levels and blood parasitemia as previously described in other tissues such as spleen or bone marrow [36].

## Conclusions

This study reports a significant reduction in *L. infantum* antibodies measured in treated dogs by an end point sera dilution ELISA after 30 days of treatment associated with clinical improvement. A low number of sick dogs with moderate disease was negative by blood real-time PCR at the time of diagnosis.

## Abbreviations

EU: ELISA units
IQR: interquartile range
PBS: phosphate buffered saline
PCV: packed cell volume
r_s_: Spearman's correlation coefficient
SD: standard deviation
UPC: urinary protein creatinine ratio
